# Insulin-like growth factor binding protein 2: a core biomarker of left ventricular dysfunction in dilated cardiomyopathy

**DOI:** 10.1186/s41065-023-00298-5

**Published:** 2023-10-31

**Authors:** Wei Yu, Hongli Gao, Tianyang Hu, Xingling Tan, Yiheng Liu, Hongli Liu, Siming He, Zijun Chen, Sheng Guo, Jing Huang

**Affiliations:** 1 Department of Cardiology, The Yongchuan Hospital of Chongqing Medical University, Chongqing, China; 2 Department of Cardiology, The Second Affiliated Hospital of Chongqing Medical University, Chongqing, China; 3 Precision Medicine Center, The Second Affiliated Hospital of Chongqing Medical University, Chongqing, China; 4 Department of Cardiology, The People’s Hospital of Rongchang District, Chongqing, China

**Keywords:** Dilated cardiomyopathy, RNA modifications, Machine learning, IGFBP2, Left ventricular ejection fraction

## Abstract

**Background:**

RNA modifications, especially N6-methyladenosine, N1-methyladenosine and 5–methylcytosine, play an important role in the progression of cardiovascular disease. However, its regulatory function in dilated cardiomyopathy (DCM) remains to be undefined.

**Methods:**

In the study, key RNA modification regulators (RMRs) were screened by three machine learning models. Subsequently, a risk prediction model for DCM was developed and validated based on these important genes, and the diagnostic efficiency of these genes was assessed. Meanwhile, the relevance of these genes to clinical traits was explored. In both animal models and human subjects, the gene with the strongest connection was confirmed. The expression patterns of important genes were investigated using single-cell analysis.

**Results:**

A total of 4 key RMRs were identified. The risk prediction models were constructed basing on these genes which showed a good accuracy and sensitivity in both the training and test set. Correlation analysis showed that insulin-like growth factor binding protein 2 (IGFBP2) had the highest correlation with left ventricular ejection fraction (LVEF) (R = -0.49, *P* = 0.00039). Further validation expression level of IGFBP2 indicated that this gene was significantly upregulated in DCM animal models and patients, and correlation analysis validation showed a significant negative correlation between IGFBP2 and LVEF (R = -0.87; *P* = 6*10^–5^). Single-cell analysis revealed that this gene was mainly expressed in endothelial cells.

**Conclusion:**

In conclusion, IGFBP2 is an important biomarker of left ventricular dysfunction in DCM. Future clinical applications could possibly use it as a possible therapeutic target.

## Introduction

Dilated cardiomyopathy (DCM), characterized by ventricular enlargement and myocardial systolic dysfunction, is one of the most intractable diseases in the cardiovascular field [1, 2]. DCM may gradually worsen into severe congestive heart failure, which poses a substantial threat to the patients' survival [3]. However, because its etiology and underlying mechanisms are not clear, existing treatment strategies, with the exception of heart transplantation, are not effective in treating DCM [4]. Therefore, identifying the specific molecular mechanisms associated with DCM is essential to prevent its poor prognosis.

Epigenetic modifications usually occur at the genomic and transcriptional levels, they also play a critical role in post-transcriptional regulation at the same time. It is characterized by changes in the temporal and spatial expression patterns of chromatin and genes driven by specific enzymes, without altering the nucleotide sequence of DNA and subsequent functional changes in genetic genes [5, 6]. RNA modification, also known as epitranscriptional modification, is one of these epigenetic modifications. To date, more than one hundred RNA modifications have been identified [7]. The process of RNA modification is reversible and it is controlled by “writers”, “readers”, and “erasers” [8]. This process can directly affect gene expression by controlling RNA processing, localization, translation, and decay, and can also affect the chemical properties of RNA, including base pairing, secondary structure, and interaction with proteins [9]. Recently, a wide range of RNA modifications, including N6-methyladenosine (m^6^A), N1-methyladenosine (m^1^A) and 5–methylcytosine(m^5^C) have been identified in cardiovascular diseases (CVDs), and these modifications are involved in the development of CVDs [10]. In addition, some studies have shown that m^6^A, m^1^A and m^5^C contribute to the progression of several pathological conditions in CVDs [11–13]. These pathophysiological changes include cardiac remodeling, myocardial fibrosis, mitochondrial dysfunction, cardiac hypertrophy and so on [14], which coincide with the pathophysiological features of DCM [15–18]. Despite this, very few studies have focused on the role of m^6^A-, m^1^A- and m^5^C-related regulators in DCM. It is currently unclear if dysregulation of the expression of these regulators contributes to the progression of DCM by inducing RNA modifications and subsequent pathophysiological alterations in the disease.

Therefore, in this study, we sought to use bioinformatics methods to explore the potential roles of m^6^A, m^1^A and m^5^C in DCM. We comprehensively searched m^6^A-, m^1^A- and m^5^C-related regulators from the published papers and obtained 45 RNA modification regulators (RMRs) with different functions [19–21]. Three machine learning methods were used to screen key RMRs for subsequent analysis. Moreover, we evaluated the correlations between key RMRs and clinical characteristics which included left ventricular ejection fraction (LVEF), left ventricular internal diastolic dimension (LVIDD), infection indexes, body mass index (BMI), gender, age, and virus infection status and inflammatory markers. Insulin-like growth factor binding protein 2 (IGFBP2) showed the highest correlation with LVEF in correlation analyses. IGFBP2 is a member of the IGFBP family and also acts as a regulator signal transducer ("reader") for m^6^A controlling its alteration [22]. Several studies have identified this gene as an important biomarker for cardiovascular disease [23–25], with a diagnostic efficacy even higher than that of B-type natriuretic peptide [25]. However, its regulatory role in DCM has been less well studied, especially the correlation between this gene and clinical traits in DCM patients. Therefore, we further validated its expression level and correlation with clinical characteristics. Finally, through single-cell analysis we identified the cell types where IGFBP2 is mainly expressed, which provides a novel perspective for the subsequent study of the regulatory mechanism of this gene.

## Materials and methods

### Acquisition and processing of datasets

GSE17800 was sourced from the NCBI Gene Expression Omnibus (https://www.ncbi.nlm.nih.gov/geo/), which contained 8 normal samples and 40 DCM patients. After downloading, the dataset was pre-processed by R and Perl, which included background calibration, normalization, and log2 transformation [26]. In addition, we identified 45 RMRs by reviewing the literature and obtained the expression of RMRs by the “LIMMA” package in the DCM dataset for subsequent analysis of differential expression of RMRs [27]. Another independent dataset (GSE116250) for subsequent validation of the model and diagnostic efficacy of key genes. GSE145154 was used as a single-cell set.

### Functional enrichment analysis

Gene ontology (GO) and Kyoto Encyclopedia of Genes and Genomes (KEGG) pathways were completed by the " clusterProfiler" R package to analyze the functional pathways enriched by differentially expressed RMRs (DE-RMRs) [28]. Disease ontology (DO) enrichment analysis was completed on these genes via the “clusterProfiler” package and DOSE package in R [29]. GO/DO terms and KEGG pathways with *p*-value < 0.05 were considered to be statistically significant.

### Screening of key RNA Modification Regulators (RMRs)

Three machine learning methods, including least absolute shrinkage and selection operator (LASSO), support vector machine- recursive feature elimination (SVM- RFE), and random forest (RF), were used to determine key RMRs. The LASSO algorithm is a form of regression analysis that applies regularization techniques to enhance prediction accuracy [30]. We completed the LASSO regression approach in R using the "glmnet" package to reduce the dimensionality of the data to identify the genetic biomarkers related to DCM [30, 31]. SVM-RFE models were built with the “kernlab” package and compared by their tenfold cross-validated mean errors, and the gene corresponding to the minimum error point was selected as the disease signature gene [32]. RF is a popular machine learning technique that has found success in numerous industries. It is capable of creating highly accurate predictive models with minimal model optimization requirements, which makes it a valuable tool [33]. The “randomForest” package [34] was applied to generate random forest models of differentially expressed RMRs, and the decreasing accuracy approach (Gini coefficient method) was utilized to estimate the importance value of the variables of the RF model. Genes with top 8 importance values were used for further analysis. Finally, the key RMRs were obtained by taking the intersection of the genes screened by the three machine learning methods.

### Constructing risk prediction model and assessing diagnostic efficacy based on key genes

In order to forecast the likelihood of DCM, we employed a multivariate logistic regression analysis by utilizing the "rms" package in R [35]. This analysis was performed to create a predictive model based on significant genes that were identified by machine learning techniques. To assess the precision and usefulness of the nomogram, calibration and ROC curves were generated. Furthermore, in the training group, we plotted the ROC curves of these genes to evaluate their diagnostic capabilities. In the test group, we initially analyzed the expression of each gene in DCM patients and controls, and then confirmed the accuracy of the model and the diagnostic efficiency of each gene.

### Correlation analysis of key RMRs with clinical characteristics

To investigate the relationship between key genes identified by three machine learning approaches and DCM progression, we correlated these genes with clinical characteristics, which included LVEF, LVIDD, BMI, gender, age, viral infection status, and inflammatory markers (Table 1). Correlation analysis was performed using the Sperman method, and correlation coefficients (R) > 0.3 and *p*-value < 0.05 were considered as screening conditions.

**Table 1:** The clinical characteristics of GSE17800 and recruited subjects

Type	Parameter	Control	DCM	*p*-value
GSE17800	Number	*n* = 8	*n* = 40	NA
	LVEF (%)	60 (52, 64)	35 (28, 39)	< 0.001
	LVIDD (mm)	51.4 ± 3.1	69.8 ± 8.0	< 0.001
	Gender (female)	2 (25%)	12 (30%)	1.0
	Age (years)	44 (30, 57)	52 (42, 58)	0.213
	Virus (negative)	8 (100%)	18 (45%)	0.005
	Inflammation	10 (8, 13)	18 (12, 22)	0.001
	BMI (Kg/m^2^)	25.0 (23.1, 29.3)	27 (24, 30)	0.306
Recruited subjects	Number	*n* = 6	*n* = 8	NA
	LVEDV (ml)	87.4 ± 14.6	248.3 ± 81.4	< 0.001
	LVESV (ml)	29.7 ± 7.1	177.6 ± 71.0	< 0.001
	EF (%)	66.5 ± 2.9	30.2 ± 14.3	< 0.001
	LVIDD (mm)	45.0 ± 4.0	70.5 ± 8.7	< 0.001
	Age	54.6 ± 10.8	57.9 ± 8.8	0.579
	Gender (Male)	2 (40%)	5 (55.6%)	1.0

*Abbreviation*: *BMI* body mass index, *LVEF* left ventricular ejection fraction, *LVIDD* left ventricular internal diastolic dimension. *LVEDV* left ventricular end-diastolic volume, *LVESV* left ventricular end-systolic volume

### Validation of IGFBP2 expression in DCM animal models and humans

We focused on IGFBP2 due to its high correlation with clinical characteristics and validated its expression in both a DCM rat model and patients. Eight SD rats were randomly divided into two groups and given doxorubicin (HY-15142; MedChemExpress; 4 mg/kg) and saline, respectively, once a week for 8 weeks. After 8 weeks, we evaluated cardiac function in the rat using ultrasound, measuring left ventricular end-diastolic volume (LVEDV), LVEF, LVIDD, and left ventricular end-systolic volume (LVESV). The observed progressive decline in cardiac function and ventricular dilatation indicated a successful construction of the model. The total RNA from the left ventricle was extracted using the extraction kit (NO.RE-03014, Foregene), followed by reverse transcription into cDNA using PrimeScrip RT Master Mix (RR036A, Takara). Amplification was performed using SYBR Green PreMix (RR420A, Takara), and qPCR was carried out with GAPDH as the internal control. The primer sequences of the studied genes are as follows: (forward primer) 5′-ACAGCAACAGGGTGGTGGAC-3′ and (reverse primer) 5′-TTTGAGGGTGCAGCGAACTT-3′ for GAPDH; (forward primer) 5′-TGGACGGAACCATGAACATG-3′ and (reverse primer) 5′-ACACAGCCAGTTCCTTCATG -3′ for IGFBP2. All animal experiments in this study were approved by the Institutional Animal Ethics Committee of the Second Affiliated Hospital of Chongqing Medical University (permit number: (2021)347) and conducted in accordance with the relevant guidelines.

Meanwhile, eight patients with dilated cardiomyopathy and six age-matched healthy Individuals were recruited in the yongchuan Hospital affiliated of chongqing medical university from february to april 2023. The diagnosis of DCM follows relevant guidelines [36]: left ventricular or biventricular dilatation and systolic dysfunction, while excluding that these changes are caused by hypertension, valvular or coronary artery disease. We recruited six healthy individuals from the medical examination center at the hospital. Our study adhered to the principles outlined in the Helsinki Declaration and received approval from the Hospital Medical Ethics Committee (permit number: 2019029). All participants received written informed consent. The study involved collection of venous blood from both cases and controls, which was then subjected to centrifugation to separate the serum. Subsequently, the IGFBP2 content in all serum was detected by ELISA (E-EL-H6038, Elabscience). Finally, we collected echocardiographic data including LVEDV, LVESV, LVEF, and LVIDD from the patients and verified the correlation between IGFBP2 concentrations in serum and LVEF and LVIDD using the Sperman method.

### Single cell analysis

The regulatory pattern of IGFBP2 was explored at the single cell level. Quality control, dimensionality reduction and clustering of DCM scRNA-seq data were performed using “Seurat” package, as described in previous studies [37]. “singleR” R package was used to annotate the clusters, and then “CellMarker” was used for manual correction [38]. Based on the identified single-cell clusters, we further evaluated the expression of IGFBP2 in each cell subset. Integration of all single-cell rank-based gene set enrichment analyses with the hallmark gene sets was performed by irGSEA (version 2.1.4) R package.

### Statistical analysis

The statistical analysis was conducted using SPSS (version 26.0) and R (version 4.3.0) software. Continuous variables were analyzed using either the independent sample t-test or Mann–Whitney U test depending on their normality of distribution. The Wilcoxon sum-rank test or chi-square test was applied to analyze categorical variables. We employed the Spearman method to test the correlation between key RMRs and clinical characteristics. A *p*-value of less than 0.05 was used to indicate statistical significance.

## Results

### Identification of key RMRs in dilated cardiomyopathy

**Fig. 1:**
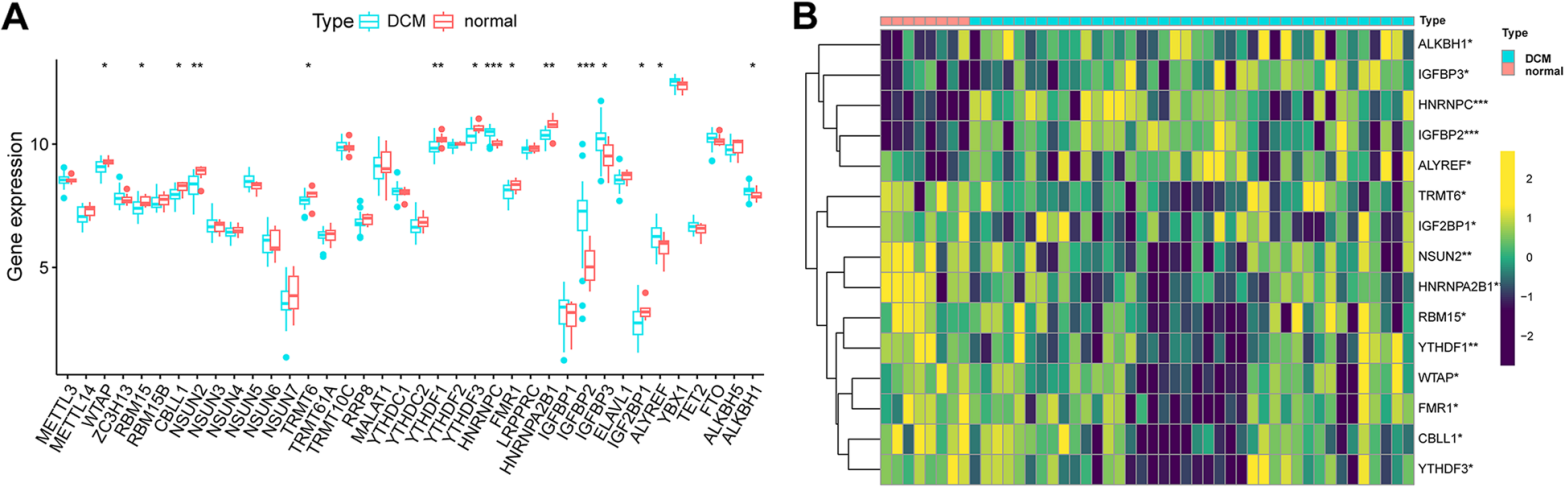
Differential expression analysis of RNA modification regulators (RMRs) in DCM. **A** Expression of RMRs in normal group versus DCM group. **B** The heatmap of RMRs between normal samples and DCM samples. Blue represents DCM group and red represents normal group. DCM, dilated cardiomyopathy. RMRs, RNA modification regulators; * represents *p* < 0.05; ** represents: *p* < 0.01; *** represents: *p* < 0.001

**Fig. 2:**
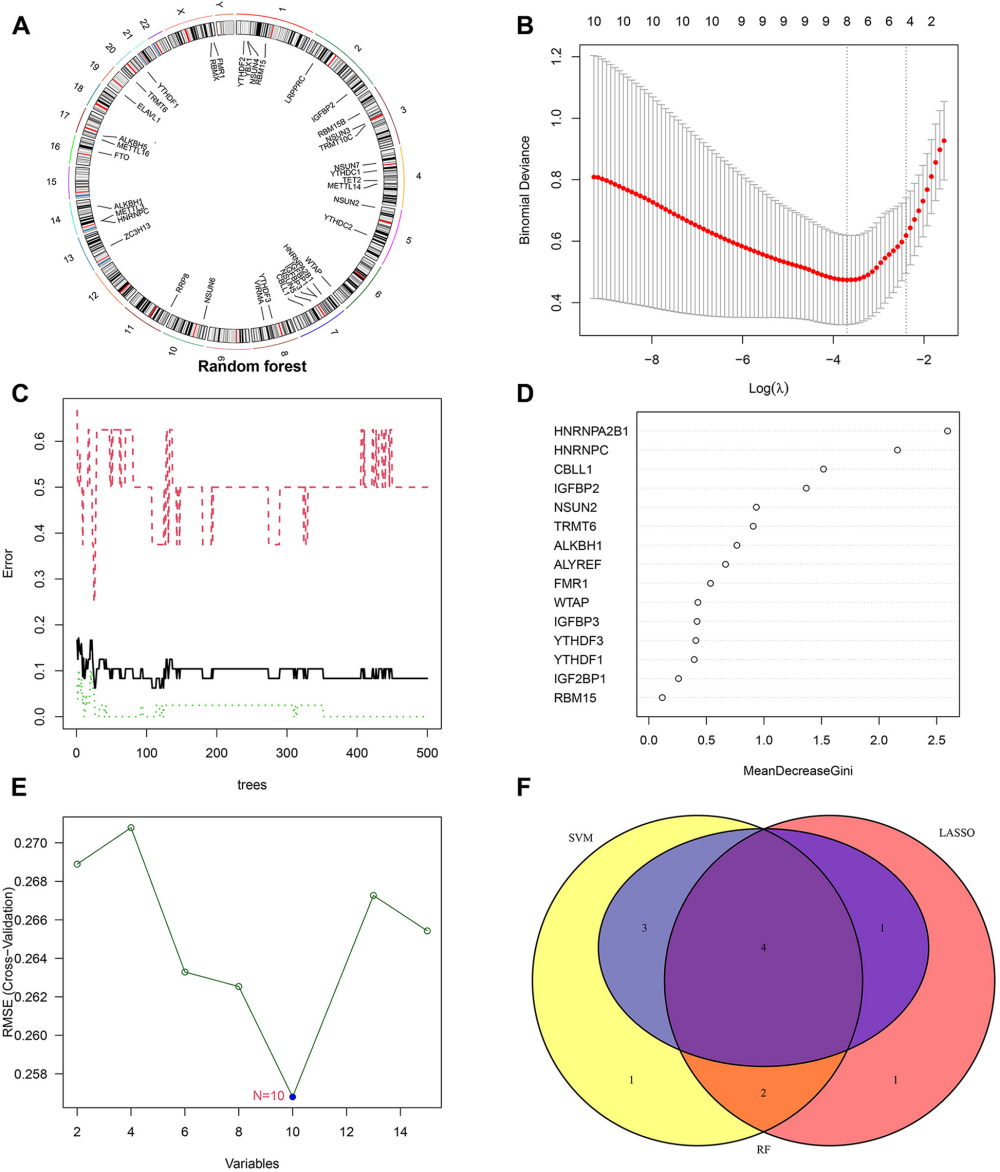
Screening for key RNA modification regulators (RMRs) in DCM. **A** Circle diagram of RMRs at different chromosomal locations. **B** Tuning feature screening in the LASSO model. **C** Curve of error versus number of decision trees. Red represents the DCM group, green represents the control group, and black represents all of them. **D** Results of the Gini coefficient method in random forest classifier. The x-axis indicates the genetic variable, and the y-axis represents the importance index. **E** Screening plot of key RMRs based on SVM-RFE algorithm. **F** The Venn diagram showing the 4 RMRs shared by LASSO, SVM-RFE and RF

First, we extracted the expression of 45 RMRs in the train group and performed differential expression analysis. Differential expression analysis revealed significant differences in the expression levels of 15 RMRs between the DCM and control groups (Fig. 1A and B). And the location of RMRs on different chromosomes was illustrated in Fig. 2A. Subsequently, three different machine learning approaches were used to screen for hub genes in DE-RMRs. Eight variables were identified as Characteristic genes of DCM from DE-RMRs by the LASSO regression algorithm (Fig. 2B). We used 15 DE-RMRs to train a random forest classifier. To identify the optimal parameters for the recurrent random forest classification, we evaluated the model's error rate. Based on the graph of the model error versus the number of decision trees (Fig. 2C), we selected 26 trees as the parameters for the final model, which demonstrated a stable model error. Subsequently, we measured the variable importance of the output in terms of decreasing precision and decreasing mean square error (Gini coefficient method) and plotted the importance scores of the variables (Fig. 2D). The top 8 genes were chosen as candidate genes for further study. Moreover, we used the SVM-RFE algorithm to identify the feature DE-RMRs. The results revealed that the model had the lowest error when N = 10 implying that all DE-RMRs were included (Fig. 2E). The intersection of genes identified by the three machine learning methods revealed four key RMRs (Fig. 2F).

### Enrichment analyses of the RMRs

**Fig. 3:**
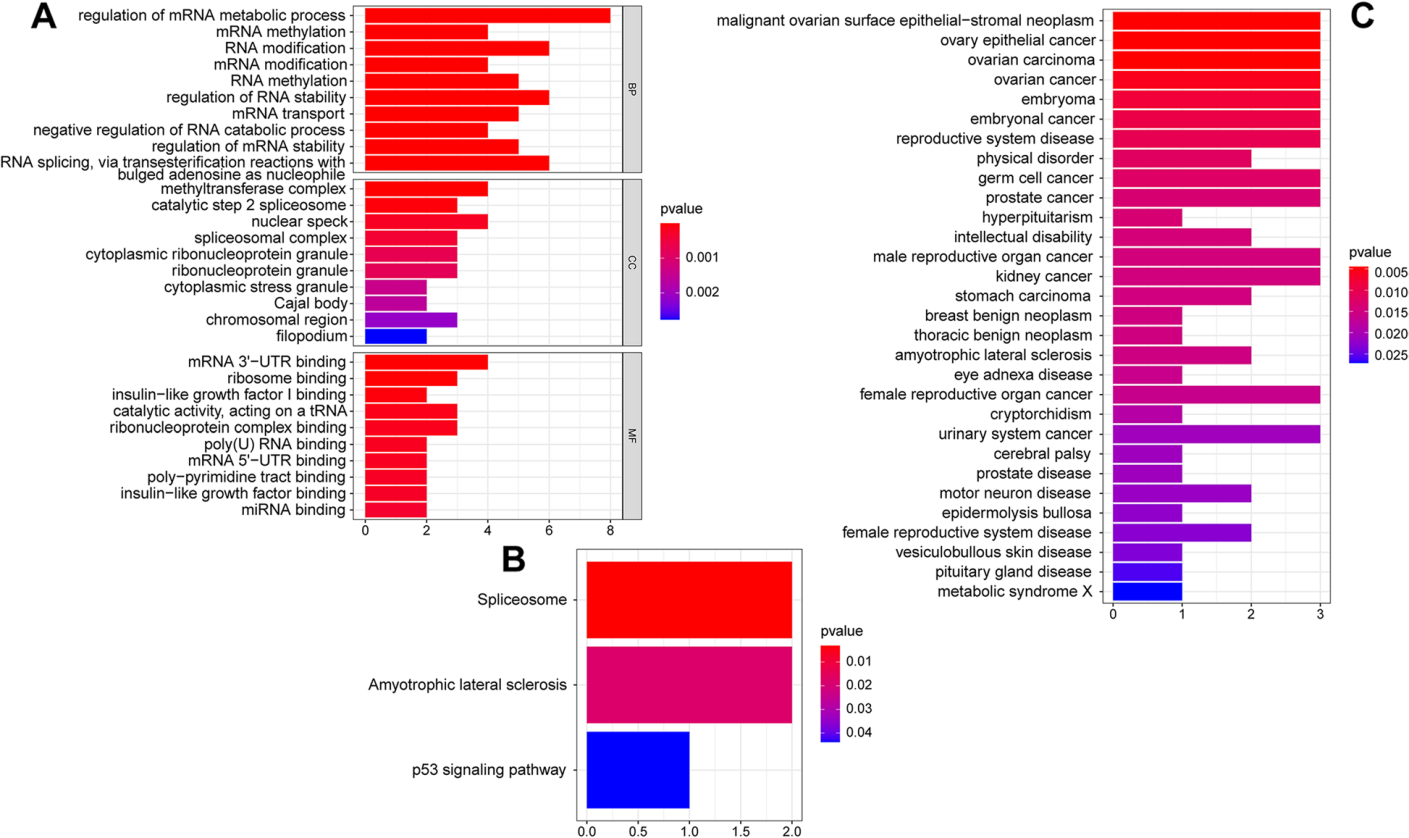
Gene enrichment analysis of differentially expressed RNA modification regulators (DE-RMRs). **A** GO enrichment analysis of DE-RMRs. **B** KEGG pathway analysis of DE-RMRs. **C** DO enrichment analysis of DE-RMRs. Abbreviations: GO, Gene Ontology; KEGG, Kyoto Encyclopedia of Genes and Genomes; DO, Disease Ontology; DE-RMRs, differentially expressed RNA modification regulators

GO, KEGG, and DO analyses were carried out to identify the biological processes and pathways related to RMRs. According to the results of GO analysis, DE-RMRs were mainly involved in regulation of mRNA metabolic process, RNA modification, methyltransferase complex, mRNA 3' − UTR binding and ribosome binding (Fig. 3A). In the KEGG pathway, DE-RMRs were mainly involved in the Spliceosome, Amyotrophic lateral sclerosis and p53 signaling pathway (Fig. 3B). In DO analysis, DE-RMRs were mainly involved in malignant ovarian surface epithelial − stromal neoplasm, ovary epithelial cancer and embryoma (Fig. 3C).

### Construction of model and evaluation of genetic diagnostic efficacy

**Fig. 4:**
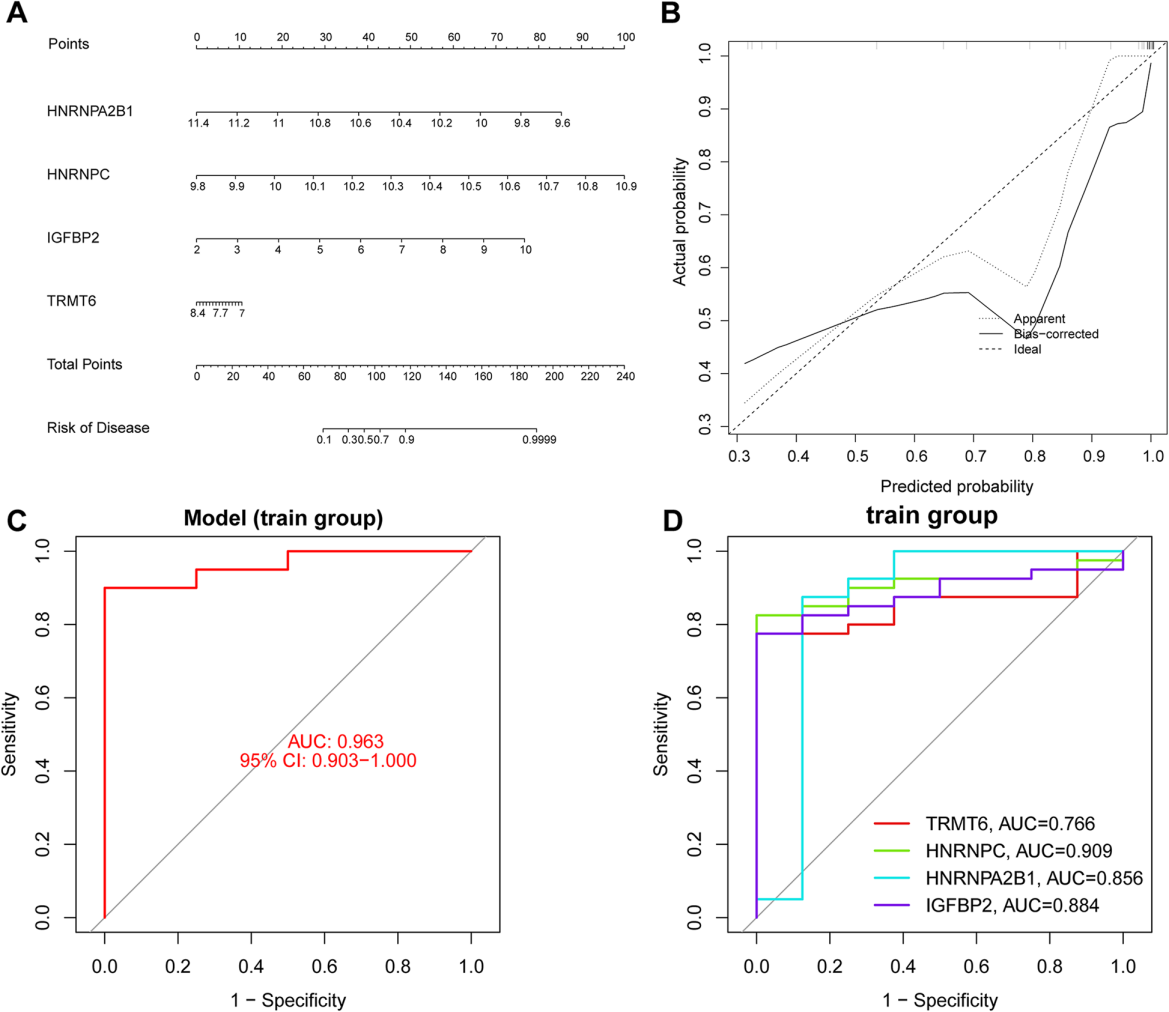
Construction and validation of nomogram. **A** Development of a prediction nomogram based on four key RMRs. **B** Calibration curves for predicting risk of dilated cardiomyopathy nomogram. **C** ROC curve of risk prediction model in the train group (GSE17800). **D** Assessment of the diagnostic efficacy of key RMRs in the train group

**Fig. 5:**
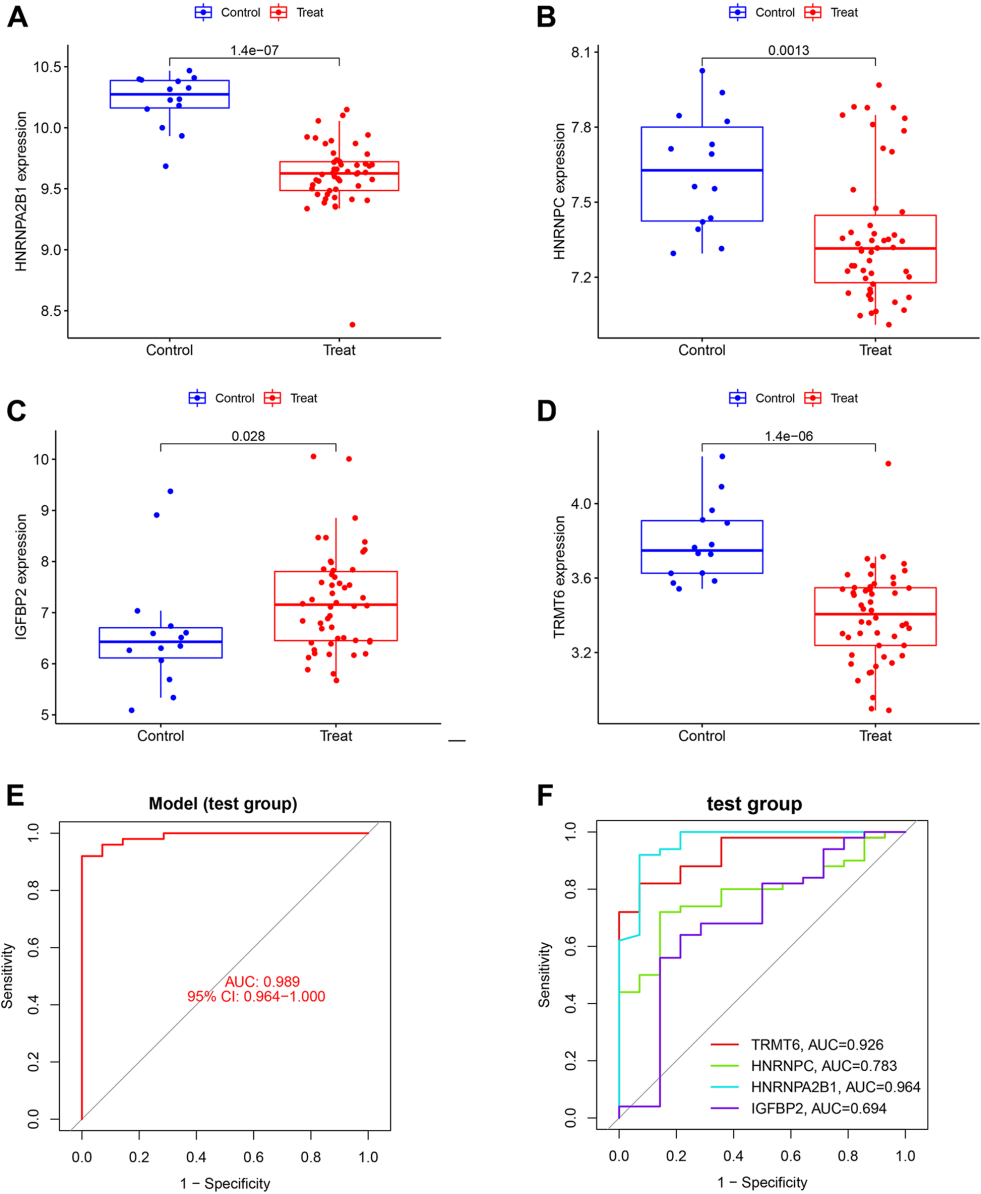
Assessment of gene expression and diagnostic efficacy in test group. Gene expression levels of (**A**) HNRNPA2B1, (**B**) HNRNPC, (**C**) IGFBP2 and (**D**) TRMT6 between normal and DCM samples in the test group. (**E**) ROC curve of risk prediction model in the test group (GSE116250). **F** Assessment of the diagnostic efficacy of key RMRs in the test group

We conducted multifactorial logistic regression analysis on the screened RMRs and developed a nomogram to predict the risk of DCM (Fig. 4A). We also generated calibration curves to assess the performance of the nomogram, which demonstrated good agreement (Fig. 4B). Furthermore, the ROC curve for the risk prediction model showed an impressive AUC of 0.963 (Fig. 4C), indicating high accuracy and sensitivity of the model. In addition, in the train group, these four genes also showed good diagnostic capability to differentiate between DCM patients and normal individuals (Fig. 4D). In the test group, we further evaluated the expression of these genes and found that they were significantly different between DCM patients and normal samples (Fig. 5A-D). Further, the risk prediction models had good accuracy and sensitivity in the train group (Fig. 5E), and these genes also had good diagnostic efficacy (Fig. 5F).

### Correlation analysis of key RMRs with clinical characteristics

**Fig. 6:**
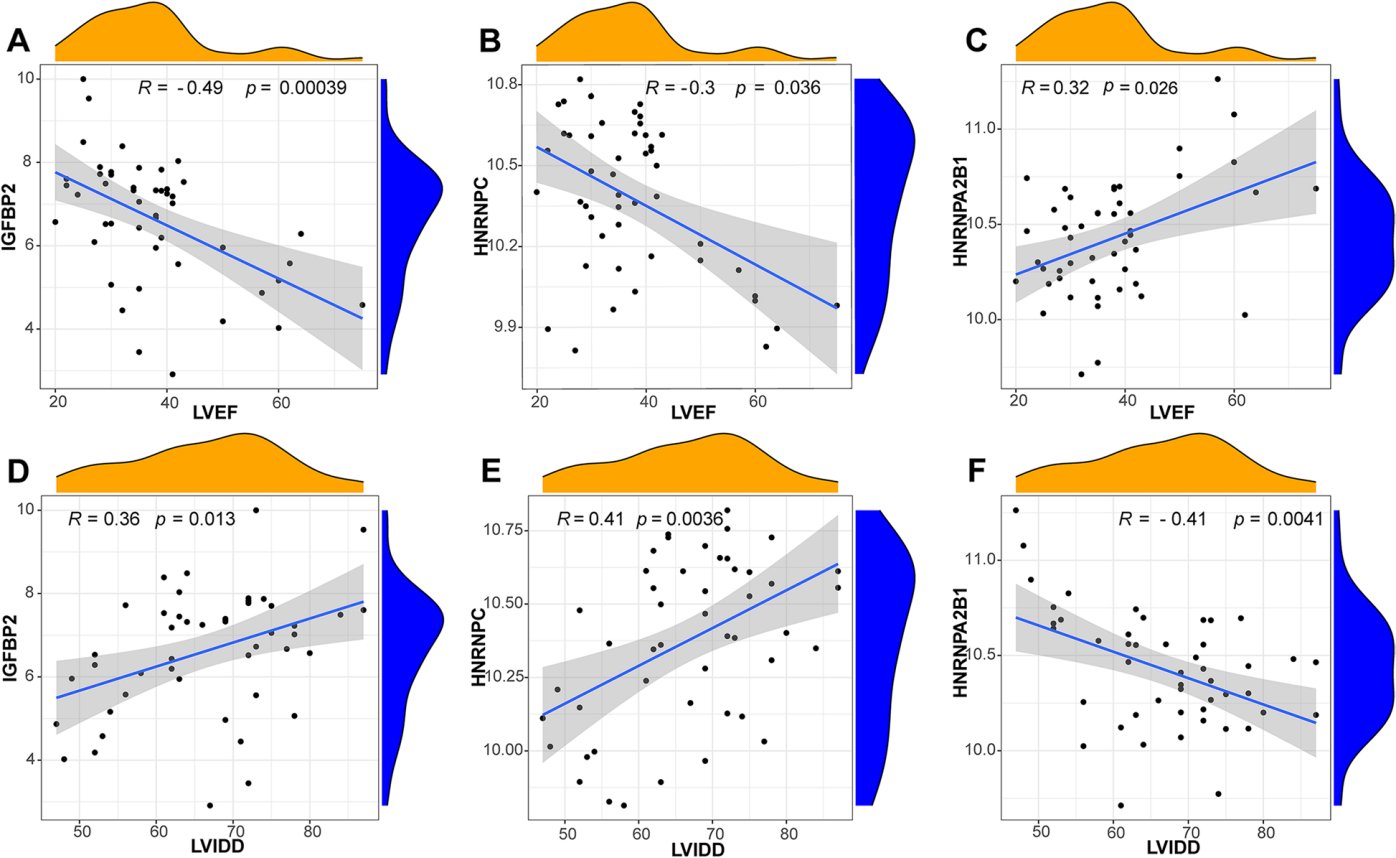
Correlation analysis of 4 key RMRs with clinical characteristics. Correlation analysis of IGFBP2 (**A**), HNRNPC (**B**) and HNRNPA2B1 (**C**) with LVEF. Correlation analysis of IGFBP2 (**D**), HNRNPC (**E**) and HNRNPA2B1 **F** with LVIDD. LVEF, left ventricular ejection fraction; LVIDD, left ventricular internal diastolic dimension

We further analyzed the correlation of 4 key RMRs with clinical characteristics to understand the impact of these genes on DCM progression. From the results, IGFBP2 (R = -0.49; *p* = 0.00039) and HNRNPC (R = -0.3; *p* = 0.036) were negatively correlated with LVEF (Fig. 6A and B), while HNRNPA2B1 (R = 0.32; *p* = 0.026) was positively correlated with LVEF (Fig. 6C). In addition, IGFBP2 (R = 0.36; *p* = 0.013) and HNRNPC (R = 0.41; *p* = 0.0036) were positively correlated with LVIDD (Fig. 6D and E), while HNRNPA2B1 (R = -0.41; *p* = 0.0041) was negatively correlated with LVIDD (Fig. 6F). These results reveal, to some extent, the effect of these genes on the development of DCM. In addition, based on the highest correlation between IGFBP2 and LVEF in the correlation analysis, further analysis and validation of this gene was subsequently performed.

### Validation of IGFBP2 expression in animals and humans

**Fig. 7:**
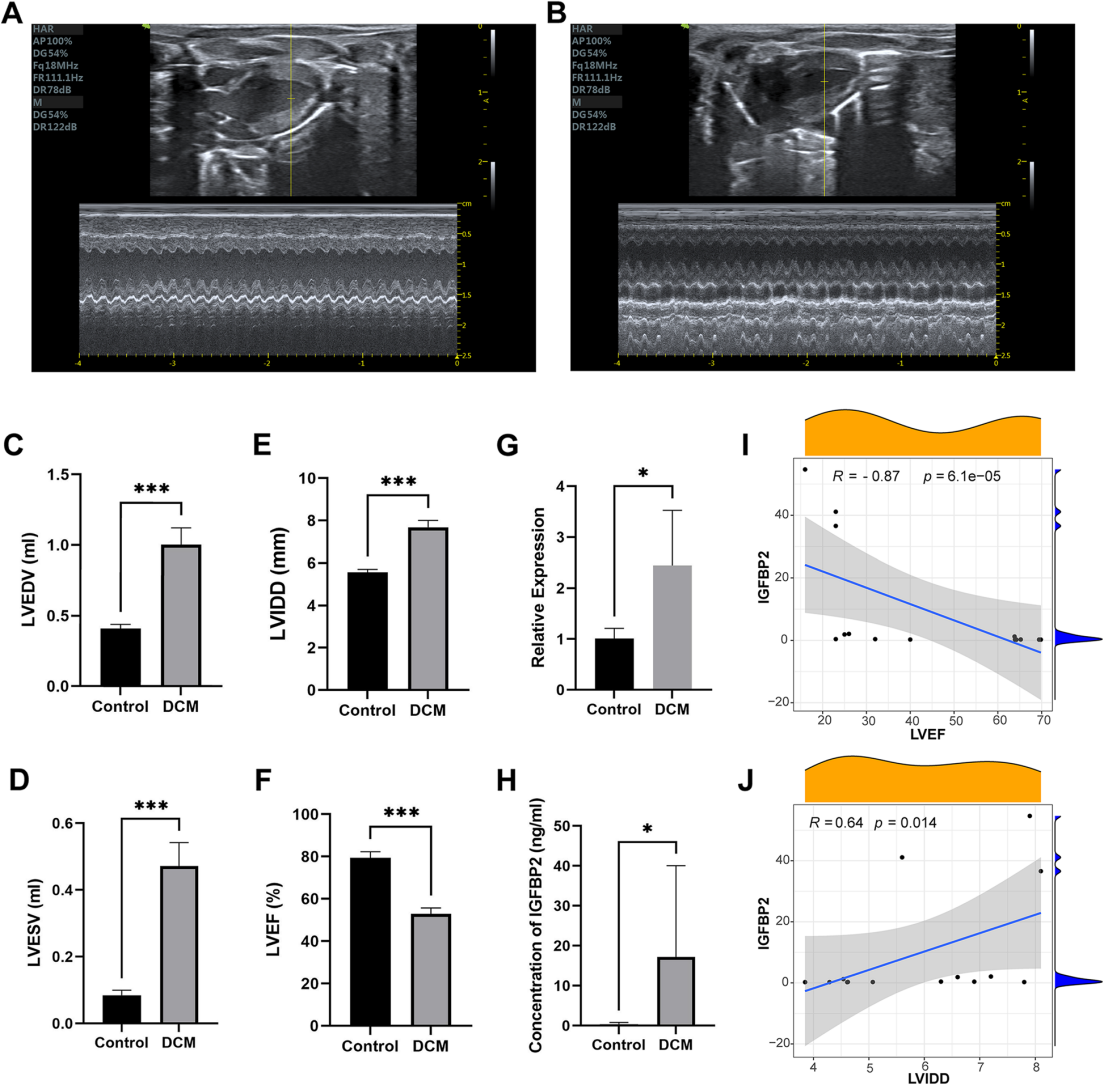
Validation of IGFBP2 expression. Echocardiography of rats in DCM (**A**) and control (**B**) groups. **C** LVEDV,** D** LVESV, **E** LVIDD, **F** LVEF of rats treated with saline or doxorubicin. **G** Relative expression of IGFBP2 in the hearts of rats treated with doxorubicin, normalized to the expression of GAPDH. **H** Measurement of IGFBP2 expression in serum of DCM patients and normal individuals by ELISA. **I** Correlation of IGFBP2 with LVEF. **J** Correlation of IGFBP2 with LVIDD. * represents: *p* < 0.05; *** represents: *p* < 0.001

After 8 weeks of DOX administration, rats in the DCM group exhibited reduced left ventricular wall beat amplitude, ventricular dilatation, and left ventricular systolic dysfunction as observed through ultrasonography (Fig. 7A-F). The expression of IGFBP2 was significantly upregulated in the DCM group compared to the control group (Fig. 7G). Our findings were further validated in humans, where the concentration of IGFBP2 in the serum of DCM patients was significantly higher than that in the control group (Fig. 7H). We verified the correlation between serum IGFBP2 concentration and LVEF and LVIDD (Fig. 7I and J), and we found that IGFBP2 was significantly negatively correlated with LVEF (R = -0.87; *p* = 6*10^–5^) and positively correlated with LVIDD (R = 0.64; *p* = 0.014), which was consistent with the results obtained in the dataset.

### Single-cell analysis

**Fig. 8:**
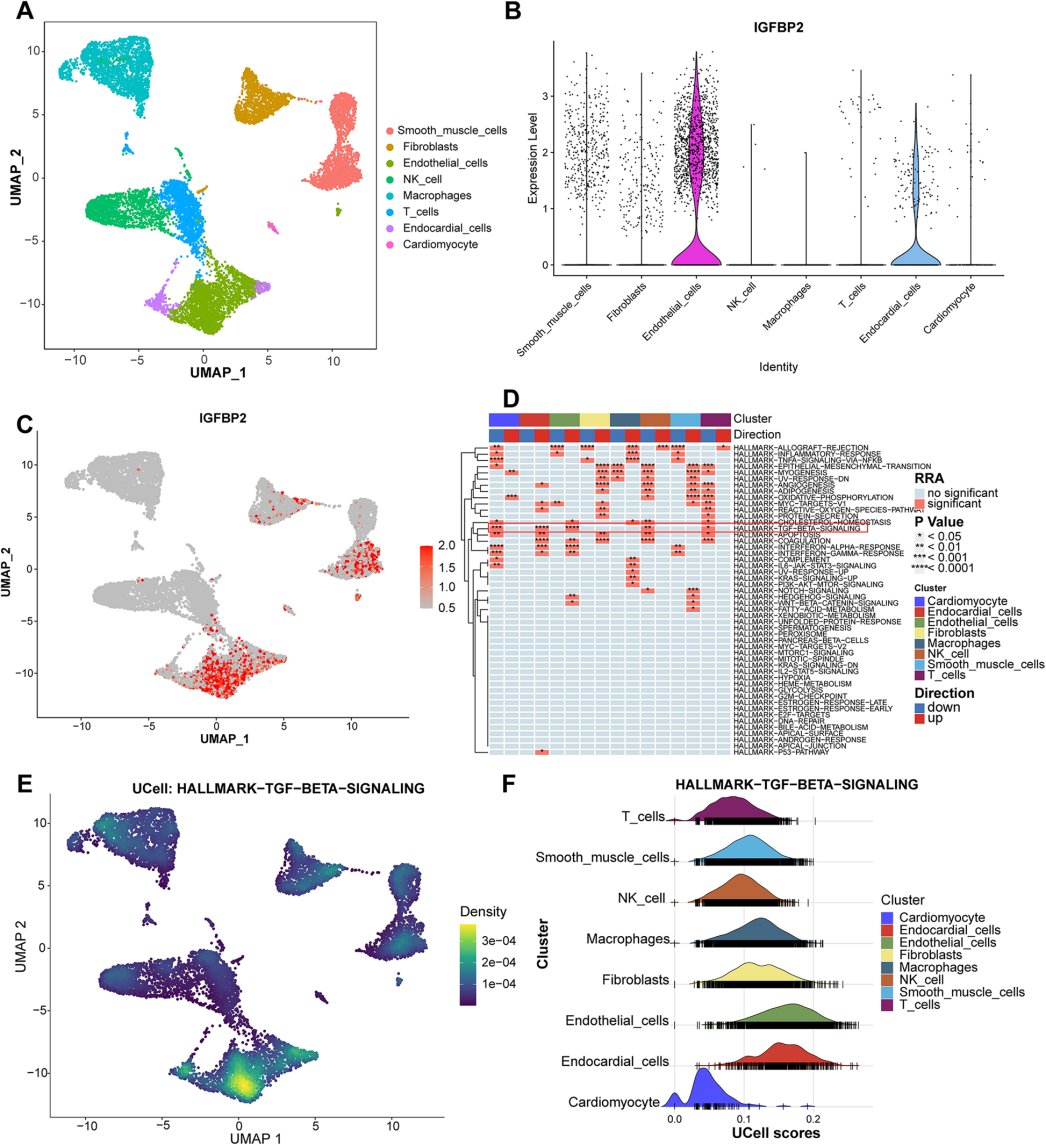
Single cell analysis of DCM patients. **A** Umap of eight cell types in GSE145154. **B** Expression of IGFBP2 in the eight cell populations. The legend shows the normalized expression of the color gradient. **C** Violin plot showing IGFBP2 expression in all cell populations. **D** Heatmap for single-cell rank-based gene set enrichment analysis. **E** Density scatterplot of TGF beta signaling. **F** Ridge plot of TGF beta signaling

Single-cell analysis was performed on left ventricular tissue from DCM patients, and a total of eight cell populations were identified by dimensionality reduction and clustering (Fig. 8A). Further analysis of the distribution of IGFBP2 in these cell populations revealed that the gene was mainly expressed in endothelial cells (Fig. 8B and C). The differential pathways of each cell cluster were further analyzed by irGSEA. The results show that endothelial cells significantly upregulate TGF beta (TGF-β) signaling (Fig. 8D-F).

## Discussion

In our study, we screened DE-RMRs between DCM patients and normal subjects by differential expression analysis. Subsequently, we used three different machine learning methods (LASSO, SVM, and RF) to identify the hub genes in DE-RMRs and used these hub genes to construct a risk prediction model for DCM. Through correlation analysis of genes and clinical traits, we found that IGFBP2 was significantly negatively correlated with LVEF (R = -0.49; *P* = 0.00039) and significantly positively correlated with LVIDD (R = 0.36; *p* = 0.013). Enrolled participants showed a significant negative association of IGFBP2 with LVEF (R = -0.87; *P* = 6*10^–5^), and a significant positive association with LVIDD (R = 0.64; *p* = 0.014). However, there are few studies focused IGFBP2 in DCM [39]. One study found that high IGFBP2 levels were associated with poorer prognosis in patients with DCM [39]. Unfortunately, this study only investigated the relationship between IGFBP2 levels and patient prognosis and did not assess the association between expression level and clinical characteristics, nor did it explored the reasons why elevated serum IGFBP2 levels led to poor prognosis. Our study identified a significant correlation between the expression level of IGFBP2 and LVEF. This result was consistent with Johanna’s study which found a significant negative correlation (R = -0.16; *P* = 0.03) between IGFBP2 and LVEF in patients with aortic stenosis (AS) [40]. Similarly, IGFBP2 levels were found to be associated with the amount of left ventricular stroke volume in patients with AS [41]. Notably, patients with decreased ejection fraction exhibit the most substantial cardiovascular mortality compared to those with preserved ejection fraction or moderately decreased ejection fraction [42]. As a result, IGFBP2 may cause an adverse prognosis by altering the left ventricular function. In our study, we also found a significant positive correlation between the expression level of IGFBP2 and LVIDD. As one of the main parameters used to evaluate left ventricular systolic function, LVIDD, its abnormality is usually associated with left ventricular dysfunction [43]. The results of the correlation analysis between IGFBP2 and LVIDD further supported that IGFBP2 is a core biomarker of left ventricular dysfunction.

Furthermore, single-cell analysis directly revealed that IGFBP2 was mainly expressed in the endothelial cells of DCM patients. In the heart, about 30% of the cells are cardiomyocytes, the remaining 70% are endothelial cells, fibroblasts, smooth muscle cells and immune cells [44]. Endothelial cells have been shown to directly modulate the contractile state of subjacent cardiomyocytes [45]. Endothelial dysfunction can lead to sluggish myocardial relaxation and left ventricular filling impairment [46–48]. In addition, patients with DCM often suffer from endothelial dysfunction [49, 50]. IGFBP2 has been shown to directly regulate endothelial cell function [51], and alterations in its balance may lead to endothelial dysfunction [52]. Thus, IGFBP2 may contribute to left ventricular dysfunction and poorer prognosis in patients with DCM by participating in endothelial cell dysfunction. Furthermore, pathway enrichment analysis on single cells has identified significant upregulation of the TGF beta (TGF-β) signalling in endothelial cells. TGF-β promotes the conversion of endothelial cells to mesenchymal cells, during which the derived myofibroblasts secrete large amounts of extracellular matrix into cardiac and perivascular tissues, leading to excessive collagen deposition [53]. The accumulation of excessive collagen disrupts myocardial structure and leads to ventricular remodeling and cardiac fibrosis [54]. Prolonged myocardial fibrosis further leads to ventricular dysfunction, reduced heart contractility and cardiac compliance [55]. IGFBP2 may promote the conversion of endothelial cells to mesenchymal cells through TGF-β signaling, thus aggravating cardiac fibrosis progression, which negatively affects the prognoses and left ventricular functions of DCM patients. Subsequently, we will further study the regulatory pattern of IGFBP2 at the animal and cellular levels.

Of course, our study had some limitations. First, our result didn’t validate in cardiomyocytes due to lack of myocardial biopsy tissue. However, to ensure the reliability of the results, further validation was performed in another independent DCM dataset, animal models and serum from DCM patients. Furthermore, we recruited a relatively small sample of patients. So based on our preliminary results, further studies are needed, especially in relation to clinical features, to understand the pattern of the effect of this gene on DCM.

## Conclusion

In summary, by three machine learning approaches, this study identified four key RNA modification regulators. Based on these regulators, we constructed and validated a risk prediction model for DCM. Furthermore, IGFBP2 had the highest correlation with LVEF in the analysis of the correlation between these genes and clinical characteristics. Further validation in recruited subjects also confirmed a higher negative correlation of IGFBP2 with LVEF and a positive correlation with LVIDD, suggesting that this gene is a key biomarker of left ventricle dysfunction in DCM patients. Single-cell analyses showed that IGFBP2 was highly expressed predominantly in endothelial cells, suggesting that this gene may contribute to left ventricular dysfunction and poor prognosis in patients with DCM by affecting endothelial cell function.

## Data Availability

The data for this study were obtained from the GEO database (ID: GSE17800, GSE116250 and GSE145154).
